# Averting BER Floor with Iterative Source and Channel Decoding for Layered Steered Space-Time Codes

**DOI:** 10.3390/s21196502

**Published:** 2021-09-29

**Authors:** Nasru Minallah, Ishtiaque Ahmed, Jaroslav Frnda, Khurram S. Khattak

**Affiliations:** 1 Department of Computer Systems Engineering, University of Engineering and Technology Peshawar, Peshawar 25000, Pakistan; n.minallah@uetpeshawar.edu.pk (N.M.); khurram.s.khattak@uetpeshawar.edu.pk (K.S.K.); 2 National Center in Big Data and Cloud Computing, University of Engineering and Technology Peshawar (NCBC-UETP), Peshawar 25000, Pakistan; 3 Department of Quantitative Methods and Economic Informatics, Faculty of Operation and Economics of Transport and Communications, University of Zilina, 01026 Žilina, Slovakia; jaroslav.frnda@fpedas.uniza.sk

**Keywords:** antenna arrays, BER floor, EXIT analysis, high efficiency video coding, iterative decoding, layered steered space-time codes

## Abstract

The widespread development in wireless technologies and the advancements in multimedia communication have brought about a positive impact on the performance of wireless transceivers. We investigate the performance of our three-stage turbo detected system using state-of-the-art high efficiency video coding (HEVC), also known as the H.265 video standard. The system makes use of sphere packing (SP) modulation with the combinational gain technique of layered steered space-time code (LSSTC). The proposed three-stage system is simulated for the correlated Rayleigh fading channel and the bit-error rate (BER) curve obtained after simulation is free of any floor formation. The system employs low complexity source-bit coding (SBC) for protecting the H.265 coded stream. An intermediate recursive unity-rate code (URC) with an infinite impulse response is employed as an inner precoder. More specifically, the URC assists in the prevention of the BER floor by distributing the information across the decoders. There is an observable gain in the BER and peak signal-to-noise ratio (PSNR) performances with the increasing value of minimum Hamming distance ($d_{H,\min}$) using the three-stage system. Convergence analysis of the proposed system is investigated through an extrinsic information transfer (EXIT) chart. Our proposed system demonstrates better performance of about 22 dB than the benchmarker utilizing LSSTC-SP for iterative source-channel detection, but without exploiting the optimized SBC schemes.

## 1. Introduction

To meet the unprecedented expansion in the demand for wireless services over the last decade, researchers need to strongly consider uplifting the communication systems [1]. It is anticipated that this trend of upsurge in wireless services will further increase, additionally demanding much higher data rates [2]. Cisco estimates that by the year 2023 [3], there will be 3.6 global devices and connections per capita with an average data rate of 110 Mbps. Therefore, novel optimal techniques providing near-capacity spectral efficiency in future wireless networks need to be constructed.

### 1.1. Related Work

Owing to the potential increase in wireless devices, the burden on available bandwidth is inevitable and therefore the compression of data is required to transmit and store expansive data. In the transmission of data, reliability at the destination is an utmost desire for suitable processing. Source codes are particularly employed for the compression of data, whereas channel codes primarily resolve emerging errors or at least assist in detecting errors for ensuring reliability [4]. According to Shannon, source and channel coding designs can be designed independently from each other under ideal but valid conditions. The chief pitfall associated with independent designs is the poor performance due to the finite length and complexity of source and channel codes deployed, deriving low target error rates [4]. The technique of joint source-channel decoding (JSCD) was formulated [5] for improvements in performance by utilizing the residual redundancy in source coded stream [6,7].

Until now, turbo codes result in the highest performance and are deemed as the best codes with little required encoding and decoding complexity [8]. The principle of JSCD was adopted in turbo coding by iteratively exchanging information between the constituent decoders and referred to as iterative source-channel decoding (ISCD) [7]. Systems with ISCD exhibited lower bit-error rate (BER) and better performance gain than the systems with non-iterative schemes [9,10,11]. Typically, the number of iterations is restricted to two or three iterations as source coding renders the data to have limited residual redundancy [12,13]. Authors in [12] demonstrated the effects of incorporating redundancy in the source coded stream and declared that the ISCD performance improves with artificial redundancy.

Contemporary cellular communication relies heavily on the multiple-input multiple-output (MIMO) technique for an enhanced performance by offering diversity and multiplexing gain improvements. Modern telecommunication is entirely based upon the mechanism of antennas to perform high speed and distant communication. One simple example of an antenna is a piece metal attached to a radio. With the aim of improving BER performance and attaining power reductions, the concept of MIMO can be viewed as an extension to smart antennas [14]. The reason for the enhanced performance of MIMO systems lies in the fact that they exploit the multipath environment of wireless channel. Coherent MIMO systems are complex in terms of channel estimation and impose throughput loss [15]. Therefore, the differential space-time spreading (DSTS) technique was designed to overcome the issue of complexity while providing diversity gain [16], however no improvement in throughput was recorded. The DSTS is an attractive alternative design despite a 3 dB performance loss in comparison to the coherent systems [14]. The DSTS symbols work differentially between successive symbols during its encoding and decoding. Orthogonal spreading codes are used to spread the differentially encoded symbols as discussed in [14]. For throughput improvements, a spatial multiplexing technique based on multi-layer design, known as Vertical-Bell Labs Layered Space-Time (V-BLAST) [17], was developed as a simpler alternative to the Diagonal-Bell Labs Layered Space-Time (D-BLAST) scheme. The D-BLAST scheme is proficient enough to approach the MIMO capacity, but it is way complicated than V-BLAST. More explicitly, the V-BLAST scheme exploits the capacity potential due to multiple transmitters by performing layer by layer detection and decoding for high-speed transmission without any additional power or bandwidth. The drawback associated with BLAST signal is that it is unable to exploit transmit diversity [15]. However, luckily, transmit diversity gain can be relished with the multiplexing techniques of space-time block codes (STBCs). Therefore, an enhanced transmit diversity gain was recorded in [18] due to the linkage of V-BLAST and STBC. Another useful technique, namely beamforming [19], effectively mitigates the multiple-access interference and multiuser interference. Beamforming guarantees high directivity and gain for the desired user by reducing the gain towards interfering users.

Sphere packing (SP) modulation provides the maximum Euclidean distance and is becoming popular due to its high achievable performance when integrated with DSTS [14]. SP modulation attains diversity gain by transmitting symbols from multiple antennas, achieving maximum coding rate due to the maximum Euclidean distance between the SP symbols [14]. The SP modulation based on two orthogonal transmit antennas of size (2 × 2) using STBCs is represented as in Equation (1).

(1) $$G_{2}\left(x_{1},x_{2}\right)=\left[\begin{array}{cc} x_{1} & x_{2}\\ x_{2}^{*} & x_{1}^{*} \end{array}\right]$$

Here, (*) represents the complex conjugate of the symbol and *G*_2_ denotes the transmitted symbols by the two transmitting antennas. The columns in Equation (1) represent the spatial dimension while temporal dimension is represented by the rows for two successive time slots. The modulated signals for transmission are chosen from the available legitimate space time signals. Moreover, SP enhances the error resilience feature of the communication system by jointly designing the complex symbols [14].

Authors in [20] investigated the performance of various source-bit coding (SBC) schemes using DSTS-SP for the iterative JSCD system. Moreover, performance improvement is observed due to DSTS-SP in turbo detection [21], irregular variable length coding [22], and self-concatenated coding [23]. Authors in [24] analyzed the performance of different error protection schemes using the H.264 and H.265 standards. The schemes were specifically designed for supporting varying resolutions and motions over the noisy channel. A gain of 3 dB was recorded for the persistent scheme using the H.265 video standard over the H.264. In [25], the effects of minimum Hamming distance ($d_{H,\min}$) was investigated for the SBC based ISCD two-stage system. An improvement in the BER performance was observed with the increase in $d_{H,\min}$ value. To meet the requirements of higher data rate, spectral efficiency, low latency, and reliability in 5G networks, authors in [26] performed a detailed comparison of the low-density parity check (LDPC) and polar codes. An interesting strategy to mitigate the distortions in video transmission with the aid of dynamic algorithm is proposed in [27]. Moreover, a new parameter is introduced in [27] to gauge the distortions in video communication system due to video encoding. Authors in [28] presented a protocol deploying turbo and systematic network coding for satellite video communication using high efficiency video coding (HEVC). Different combinations of source and channel codes are investigated in [29] to find the optimal strategies for video communication over the lossy channels. An interesting characterization approach was presented in [30] to investigate the energy requirement for the H.265 standard. An efficient algorithm adjustable to variations in power and altering network environments was presented for the H.265 video streams in [31]. To boost the quality of service for the compressed H.265 multi-user stream, reinforcement learning techniques were adopted in [32]. Table 1 shows an overview of the methods and systems investigated recently related to wireless video communication.

In this treatise, we aim to attain a combinational gain using the merits of V-BLAST, space-time codes (STCs) and beamforming. These techniques are simultaneously employed in order to enhance the reliability of compressed information at the destination with lower $E_{b}/N_{0}$ requirements. The system is referred to as layered steered space-time code (LSSTC), as demonstrated in [7,33]. We have utilized iterative decoding for enhancing the performance of our proposed three-stage LSSTC system. Moreover, we have deployed multiple antenna arrays (AAs) for boosting the data rate and enhancing the overall system’s performance as stated in [34]. STCs are often employed in scenarios having multiple transmit AAs for improving reliability [33]. The V-BLAST architecture along with the proposed turbo detected system has the capability of large spectral efficiency as mentioned in [35]. We have deployed SBC as the error protection scheme, introducing artificial redundancy in the highly compressed H.265 stream for enhancing reliability. The LSSTC system is pretty much capable of averting the BER floor formation due to the employment of low-complexity unity-rate code (URC) decoder. The BER floor is a phenomenon which occurs in error correcting codes in the form of a point beyond which further improvement in BER stops to occur for any value of launched power. This will be observed in the form of a flattened region where the BER curve does not drop down further as before. Generally, error floors of the $10^{-3}$ order are observed when there are no spreading interleavers [36]. We will be utilizing spreading interleavers in our system. Moreover, the URC operates with an infinite impulse response and uniformly distributes information across the constituent decoders, attaining refined BER after the two iterations.

### 1.2. Novelty and Contribution

Normally, resource allocation in communication networks is based on the scarcity of available bandwidth. We aim to offer a unique and effective strategy to ensure an efficient overall mobile video transmission by utilizing the H.265 source encoder. The novelty and major contributions of this research work are as follows:

- Investigating the performance of iteratively decoded LSSTC-SP scheme using state-of-the-art HEVC/H.265 source encoder.
- Quantifying the effects of SBC on the attainable performance of three-stage system via extrinsic information transfer (EXIT) charts for mobile video transmission over the correlated Rayleigh channel.
- Demonstrating the effects of $d_{H,\min}$ on the BER and peak signal-to-noise ratio (PSNR) performances of the three-stage system.

Section 2 presents an overview of the LSSTC encoding and decoding models. In Section 3, we portray the source encoding deployed and elaborate the soft-bit source decoding (SBSD) process for the generation of extrinsic information. Section 4 presents simulation results and EXIT chart analysis of the LSSTC-SP aided iteratively decoded system. A brief description of future work is presented in Section 5, followed by a succinct conclusion in Section 6.

## 2. Overview of Layered Steered Space-Time Codes

A three-stage serially concatenated iterative JSCD system is presented deploying the combinational gain technique (V-BLAST, STC and beamforming) for the H.265 video coding standard. The three-stage iterative decoding system employs two iterations between the inner SP and intermediate URC decoder and the other iteration between the outer SBSD decoder and the intermediate URC decoder.

### 2.1. Transmitter Model

**Figure 1:**
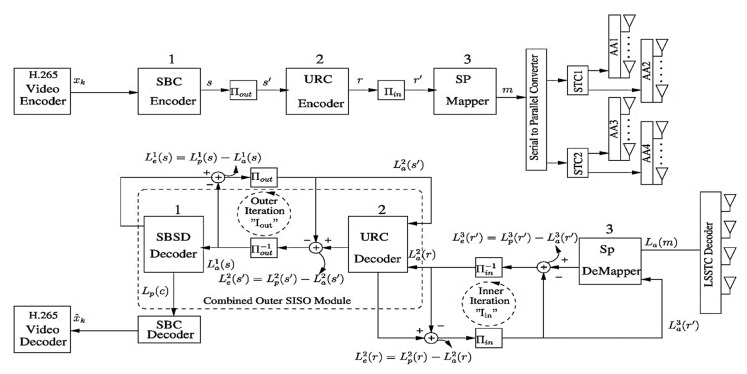
Block diagram of the three-stage LSSTC scheme with iterative JSCD system.

Figure 1 depicts the block diagram of our proposed scheme. The LSSTC architecture shown in Figure 1 utilizes $N_{t}=4$ transmit AAs. The geometry of AA is discussed in [34] for enhancing the capacity of a wireless system. For the purpose of attaining transmit diversity via independent fading experience of each AA, they are placed at sufficient distance apart from one another. Each AA contains *N* elements which are typically spaced at a distance of d=λ/2 from one another, where λ represents the carrier’s wavelength. This spacing among the *N* elements is beneficial for introducing beamforming gain. On the receiving side, there are 4 antennas which effectively receives the signal from the wireless channel. The information is serial-to-parallel converted and the resulting symbol streams are then independently allowed to the corresponding STC with multiple AAs as discussed in [33]. 

The information bits are initially encoded by the HEVC encoder, and the data are compressed to an extent suitable for limited bandwidth transmission. HEVC/H.265 encoder is the latest standard adopted by researchers across the world and approved by The International Telecommunication Union-Telecommunication (ITU-T) standardization sector. The H.265 standard was particularly developed to enhance the compression efficiency of Advanced Video Coding (AVC)/H.264 standard, with the consistent video quality in both standards [37]. It is worthy to mention that this compression efficiency is attained at the expense of increased computational techniques, rendering the source encoding more complex [38,39]. The H.265 encoder works by removing the temporal as well as spatial redundancy, hence it is regarded as a hybrid source codec [39]. Due to the higher compression techniques employed in H.265 encoding, the encoded streams are more vulnerable to the bit errors induced by channel propagation. Therefore, considerable degradation in the perceived visual quality is observed with even single-bit error. Hence, to mitigate the burst of errors in the H.265 encoded video stream, more efficient error protection and correction techniques need to be incorporated in the source encoded stream.

The source encoded stream $x_{k}$ is forwarded to the SBC encoder employing rate-1/3 which generates the stream s. The proposed low-complexity SBC encoder works perfectly well with the low-complexity STCs. An interleaver is invoked just after the SBC encoder which is labelled as $\Pi_{\mathrm{out}}$ in Figure 1, and the stream is then allowed to pass through the URC encoder [14]. Most often an interleaver is deployed before modulating the encoded stream over a wireless channel [14], and we have depicted that second interleaver as $\Pi_{\mathrm{in}}$ in Figure 1. We have used SP modulator for the purpose of exploiting the joint space-time symbols of the LSSTC scheme by efficient selection of single point [7]. Furthermore, the SP modulator is very much beneficial for the best possible minimum Euclidean distance in the real-valued space for constructing efficient error correcting codes [23]. Our system works on 4-dimemsional SP design as we have considered a scheme utilizing two STCs, namely STC1 and STC2 in Figure 1. More explicitly, the system will choose only from $L_{SP}$ number of legitimate SP mapped available vectors for transmission over the two STCs using AAs. According to [14], the SP modulator maps each of the total number of B binary coded bits such that each SP mapped symbol contains $log_{2}\left(L_{SP}\right)$ bits. In our case, $B=log_{2}\left(16\right)$ yields 4 channel coded bits for each SP symbol. Furthermore, a normalised Doppler frequency of 0.01 for the correlated Rayleigh fading channel is considered. More specifically, the correlated Rayleigh fading channel is considered useful for realistic wireless communication where multipath exist [23]. Moreover, the SP modulation is especially designed for a correlated Rayleigh fading environment.

### 2.2. Receiver Model

The received signal vector is mathematically given by *Y* = *HWX* + *n*, where *X* shows the transmitted signal vector, *H* represents the $N_{r}\times N_{t}$ matrix for a Rayleigh fading channel and *n* indicates the additive white Gaussian noise vector as discussed in [33,34]. The entries of *n* essentially have zero mean. The three-stage receiver model consists of the outer SBC decoder, the intermediate URC decoder, and the inner SP demapper. These three stages are respectively labelled as 1, 2, and 3 in Figure 1. These constituent decoders iteratively exchange information between each other in the decoding process for attaining convergence [14]. Robertson presented the logarithmic-likelihood ratios (LLRs) concept for scenarios where the constituent decoders iteratively share information with each other [40,41]. LLR is actually the logarithm of the probability ratio of a bit for its legitimate binary values. In Figure 1, the LLRs are represented with L(.) and distinguished with the superscripts 1, 2 and 3 for each decoder accordingly. Other associated decoding terminologies used are *a priori* or intrinsic Information ($I_{A}$), *a posteriori* information and Extrinsic Information ($I_{E}$) elaborated in [40,41]. We have used the subscripts of *a*, *p*, and *e* with the *L*(.) values for *a priori*, *a posteriori* and extrinsic values respectively.

The LSSTC decoder forwards the received complex symbols to the SP demapper in the LLR format [21]. The information exchange between SP demapper and URC decoder constitutes an inner iteration as shown in Figure 1. As SP assists in enhancing the diversity gain with the aid of multiple antennas, but this improvement comes at the cost of increased SP decoding complexity. The SP demapper continues to share information with the URC decoder for providing an enhanced extrinsic information to the URC decoder. Subtracting *a priori* LLR value $L_{a}$(.) from the *a posteriori* LLR value $L_{p}$(.) value results in extrinsic LLR information $L_{e}$(.) as labelled in Figure 1. $L_{p}^{2}$(*r*) values are generated with the aid of maximum a posteriori (MAP) algorithm [42] by deinterleaving $\Pi_{\mathrm{in}}^{-1}$ the $L_{e}^{3}$($r^{\prime }$) information and then allowing it to the URC decoder. With the aid of interleaver $\Pi_{\mathrm{in}}$, $L_{e}^{2}$(*r*) are converted to $L_{a}^{3}$($r^{\prime }$) and fed back to the SP demapper in next iterations.

For the outer iteration, $L_{a}$(.) values are passed through the URC decoder and the output is deinterleaved via $\Pi_{\mathrm{out}}^{-1}$. The information is then forwarded to the SBSD which yields a posteriori $L_{p}^{1}$(*s*). Subtracting the *a priori* $L_{a}^{1}$(*s*) information from $L_{p}^{1}$(*s*) generates $L_{e}^{1}$(*s*) which is interleaved via $\Pi_{\mathrm{out}}$ to generate $L_{a}^{2}$($s^{\prime }$) for feeding as a priori input to the URC decoder in next iterations. Furthermore, for our proposed design, we have considered a system iteration $I_{sys}$ to complete one cycle after one inner and two subsequent outer iterations, that is $I_{sys}=I_{in}+2I_{out}$. After one $I_{sys}$, the a posteriori $L_{p}$(c) is forwarded to the SBC decoder for generating the reconstructed bit stream ${\hat{x}}_{k}$ for feeding to the H.265 decoder. The bits from the H.265 decoder are used for performance evaluation and simulation results of our three-stage LSSTC design.

## 3. Source Encoding and Soft-Bit Source Decoding

We have used the 45 (176 × 144)-pixel quarter common intermediate format (QCIF) Akiyo video as test sequence and encoded the frames using the HM reference software for the H.265, also known as HEVC Test Model. The Akiyo video sequence is about a newscaster with few details in the backdrop and having minimal fluctuations. The Akiyo video test sequence is freely redistributed and publicly available at the Xiph.Org Foundation’s website. https://media.xiph.org/video/derf/. The rates of 15 frames-per-second (fps) and 64 kbps as the overall bitrate of Akiyo video after the encoding operation are considered for our simulations. Moreover, the H.265 standard is particularly designed for achieving the goals of parallel-processing, resilience to data-loss and coding efficiency [43], to say the least. This state-of-the-art compression standard can be extended to network interfacing and implementation, and to the compressed video layer with its high-level syntax providing encapsulation for the compressed video data [44]. Additionally, the H265 standard is useful for bit depth enhancement, configurability, and expandability of the set bits, and for multi-view and multimodal representations of 3D objects as discussed in [44]. There are some features in the H.265 standard which were also deployed in the H.264 architecture [44], namely:

- The picture parameter set used in H.264 stores shareable information which is rarely changed and is mostly used for decoding operation. The H.265 standard refers to this as the video parameter set (VPS).
- For the purpose of enabling video telephony and broadcast or streaming applications, the network abstraction layer (NAL) is designed which is an integral part of both the H.264 and H.265 standards.
- The concept of slices is adopted in both the H.264 and H.265 video standards. Slicing is normally used to avoid data losses by recovering the affected segments.
- Supplemental enhancement information (SEI) and video usability information (VUI) metadata are supported in both the H.264 and H.265 standards. These are put into the coded video as additional information and indicate the timing information, identify the colour space in the content, and provide other display related statistics.

Apart from above features, the H.265 standard also makes use of the inter/intra-prediction and 2D transform techniques, originally deployed in the H.261 standard. The inter and intra prediction techniques assist in determining a prediction residual, undergoing through block transformation, quantization, and entropy coding respectively for correcting the erroneous segments [43,44,45]. Traditionally, in all video standards, MacroBlocks (MBs) were used to enhance the performance of iteratively decoded systems by advocating larger interleaver lengths [23,45]. HEVC has replaced the MBs with Coding Tree Units (CTUs) supporting block structures ranging from (8 × 8 to 64 × 64) sizes, and sub-partitions the CTUs into Coding Tree Blocks (CTBs) [43]. Each CTB has its associated chroma and luma parts with sizes 16, 32, or 64 for providing better compression. Moreover, intra-prediction (I-slice) and inter-prediction constituting Bi-prediction (B-slice), and Previous-prediction (P-slice) are adopted in HEVC as slice segmentation techniques for mitigating the effects of errors in video transmission [39]. Slicing segments assist in recovering the affected slice using the correctly received segments but often increase the associated overhead. The H.265 video codec has the capability to support all the H.264 applications [46], although it primarily emphasizes on ultra-high-definition transmission without asking for considerable bandwidth. About 50% more compression of the image or video source was attained with the H.265 codec in comparison to the H.264, for the consistent quality [28,46]. This high compression efficiency is owing to the 35 intra prediction modes, whereas the intra prediction modes are limited to 9 in the H.264 standard. Moreover, an improved motion prediction technique is adopted in the H.265 as compared to the H.264 codec. Skip mode and direct mode estimations in the H.265 also enhance the performance in comparison to the H.264 standard.

SBSD exploits the natural redundancy to improve the convergence behavior of iterative decoding. SBSD gleans the residual redundancy for generating extrinsic LLRs, and hence beneficially transforms the coded video into short time frames, labelled as index i [7]. This SBSD operation results in significant performance improvement of the iteratively decoded system and is a modified version of the SBSD presented in [47,48]. More specifically, a parameter set $Y_{i}$ is constituted enclosing N coded patterns from each of the i video frames. The extrinsic LLR is computed for each information bit y with the help of all other instants of $Y_{i}$. Generally, the extrinsic LLR for each bit is given by Equation (2) as expressed in [7].

(2) $$L_{e}^{1}\left(y_{i,n}\;\left(\lambda \right)\right)=log\frac{\sum_{m=1,\;m\neq \lambda }^{M}P\left[y_{i,n}\;\left(m\right)|y_{i,n}\;\left(\lambda \right)=+1\right]}{\sum_{m=1,\;m\neq \lambda }^{M}P\left[y_{i,n}\;\left(m\right)|y_{i,n}\;\left(\lambda \right)=-1\right]}$$

In case the extrinsic LLRs are not known for reliabilities, we proceed with the a priori LLR-values for all such contributing bits. This interesting interplay between the extrinsic and a priori LLR-values is also visible in Figure 1, precisely from which it is inferable that these values keep on interchanging at successive iterations and time slots.

For a clearer understanding of the proposed scheme, we separately investigate the effects of various SBC schemes on the H.265 source coded stream. These SBC schemes differ only in the value of $d_{H,\min}$. We designate the SBC schemes having $d_{H,\min}\geq 2$ as the optimized SBC schemes, and hence refer to those as LSSTC-SP-URC-SBCs. For the SBC with $d_{H,\min}=1$ (un-optimized), the arrangement is referred as LSSTC-SP-URC-SBC^*^. More specifically, the SBC schemes are distinguished from each other based on the $d_{H,\min}$ value. Different SBC schemes are presented in Table 2, each having different $d_{H,\min}$ value. The SBC algorithm used here is based on the ideas in [6,49]. The ${\mathrm{SBC}}_{\left[K,N\right]}$ basically coverts the *K-bit* source symbols to N=(K+P)*-bit* information symbols. Here, *P* represents the number of redundant bits per source symbol. Each of these symbols will have a different probability of occurrence. The rationale of using SBC in the complex three-stage system is that it provides artificial redundancy in the source encoded data. The beauty of SBC is that it becomes very beneficial in the iterative decoding process and warrants an infinitesimal BER. More specifically, the convergence capability of the proposed three-stage system is enhanced with the optimized SBC schemes after multiple iterations. It is to be noted that there are three iterations in each $I_{sys}$ as discussed above.

For the LSSTC-SP-URC-${\mathrm{SBC}}_{\left[2,6\right]}^{*}$ benchmarker scheme, only those decimal symbols are included in Table 2 that have a $d_{H,\min}$ of 1 between them. The input 2-bits symbols having $d_{H,\min}$ of 1, are converted to 6-bits symbols by simply placing zeros as redundant bits. For the other schemes with $d_{H,\min}\geq 2$, the algorithm works by gradually increasing $d_{H,\min}$ with the increase in both *K* and *N* values. However, the overall code rate is kept consistent in all of the schemes. This SBC algorithm works in two steps for placing P=(m×K) redundant bits, where m≥1. In the first step, I=[(m−1)×K] redundant bits $r_{t}\left(i\right)$, such that i=1,2,…,I are appended by the addition of *K* bits (m−1) times to the original symbol. Hence, each symbol will be converted to [(m−1)×K] bits. In the second step, we calculate the last set of *K* redundant bits $r_{t}(k$) with the XOR operation of the *K* source bits, while setting $b_{t}\left(k\right)=0$ for k=1,2,…K. Mathematically,

(3) $$r_{t}\left(k\right)=\left[b_{t}\left(1\right)⊕b_{t}\left(2\right)\ldots ⊕b_{t}\left(K\right)\right]$$

for k=1,2,…K, while setting $b_{t}\left(k\right)=0$ and ⊕ represents the XOR operation in Equation (3). With this algorithm, we are able to generate specific *N-bit* codewords with $d_{H,\min}\geq 2$. The symbols are categorized according to their $d_{H,\min}$ into various LSSTC-SP-URC-SBC schemes until the highest $d_{H,\min}$ is attained for the specific rate SBC. Kliewer et al. [50] proved that for the convergence of an iteratively decoded system, the legitimate codewords should have a $d_{H,\min}$ of at least 2. As all the LSSTC-SP-URC-SBC schemes generated after the above two steps fulfil the Kliewer criterion, hence we can clearly forecast the effectiveness of these SBC schemes. Other system parameters deployed in our proposed three-stage LSSTC-SP setup are given in Table 3.

## 4. Simulation Results and EXIT Analysis

This section presents the performance analysis of the schemes in Table 2 employing $N_{t}=4$ transmit AAs and 4 receive antennas. The simulations of the proposed three-stage system are performed using the IT++ signal processing and communication library which is coded in C++. The IT++ supports several free and open-source libraries and is mainly utilized in numerical analysis, signal processing and digital communications. For the purpose of fair analysis, we have accordingly set the overall code rate of different SBC schemes to be persistent. The source encoded bits-to-SP symbols were carried using the anti-Gray mapping (AGM). For convenience, we refer to the AGM as the one which is different from Gray mapping. For enriching reliability in our experimental setup, we averaged the results after repeating the transmission experiment 160 times. It is to be noted that the SP scheme was considered for L=16 modulated symbols as in [14].

**Figure 2:**
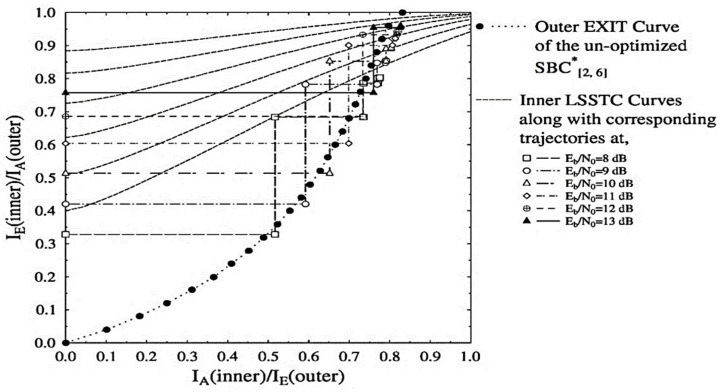
Outer and inner EXIT curves for the LSSTC-SP-URC-${\mathrm{SBC}}_{\left[2,6\right]}^{*}$ scheme along with Monte-Carlo based decoding trajectories at $E_{b}/N_{0}=$ 8 to 13 dB.

**Figure 3:**
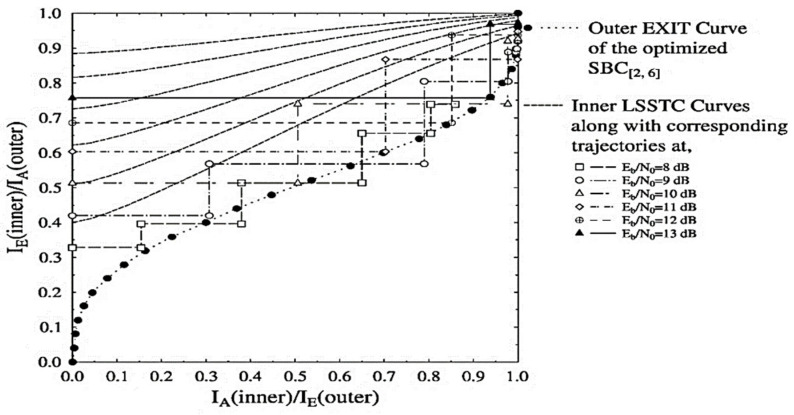
Outer and inner EXIT curves for the LSSTC-SP-URC-${\mathrm{SBC}}_{\left[2,6\right]}$ scheme along with Monte-Carlo based decoding trajectories at $E_{b}/N_{0}=$ 8 to 13 dB.

For investigating the convergence behaviour of the schemes presented in Table 2, we will be utilizing the EXIT chart analysis as proposed in [51]. EXIT chart is based on the sharing of mutual information (MI) between constituent decoders [41]. For the system proposed in Figure 1, the MI at each stage of the three decoders is distinguished from each other by replacing the subscript (.) with 1, 2, and 3 for the SBC, URC, and SP-demapper, respectively. The URC decoder yields two extrinsic outputs, labelled as $L_{e}^{2}\left(s’\right)$ and $L_{e}^{2}\left(r\right)$, and therefore its EXIT functions can be written as $F{’}_{s}\left[I_{2,A}\left(s’\right),\;I_{2,A}\left(r\right)\right]$ and $F_{r}\left[I_{2,A}\left(s’\right),\;I_{2,A}\left(r\right)\right]$ as discussed in [7]. As the SBSD and SP demapper each deal with a single input and output from and to the URC decoder, their EXIT functions are given as $F_{s}\left[I_{1,A}\left(s\right)\right]$ and $F_{r^{\prime }}\;\left[I_{3,A}\left(r’\right),\;E_{b}/N_{0}\right]$ respectively. For simplicity, we refer to the intermediate URC decoder and the SBSB as combined outer soft input soft output (SISO) module. The EXIT chart curves of the proposed SBC schemes presented in Table 2 are depicted in Figure 2 and Figure 3. It can be observed in Figure 2 that the outer EXIT curve constituting the un-optimized ${\mathrm{SBC}}_{\left[2,6\right]}^{*}$ scheme is unable to produce the required open EXIT tunnel for convergence. As all the inner LSSTC curves in Figure 2 are intersecting the outer curve prior to the (1, 1) point, it is plausible that the LSSTC-SP-URC-${\mathrm{SBC}}_{\left[2,6\right]}^{*}$ scheme is ineffective in achieving infinitesimal BER.

However, the inner LSSTC curves and the outer curve of the combined SISO module for the optimized LSSTC-SP-URC-${\mathrm{SBC}}_{\left[2,6\right]}$ arrangement succeed in attaining the (1, 1) point of perfect convergence as shown in Figure 3. As discussed above, the Kliewer criterion is met for the convergence of LSSTC-SP-URC-${\mathrm{SBC}}_{\left[2,6\right]}$ scheme. More specifically, the open EXIT tunnel between the curves provides the effective basis for attaining infinitesimal BER. Moreover, the Monte-Carlo based trajectories for the $E_{b}/N_{0}$ values of 8 to 13 dB are recorded by the exchange of MI between the inner SP demapper and the outer joint SISO module. The Monte-Carlo simulation also confirms our claim that the LSSTC-SP-URC-${\mathrm{SBC}}_{\left[2,6\right]}$ scheme is more favourable than the LSSTC-SP-URC-${\mathrm{SBC}}_{\left[2,6\right]}^{*}$ because it attains the goal of maximum extrinsic value of $I_{E}\left(outer\right)=1$ even at an $E_{b}/N_{0}$ of 1 dB, whereas the LSSTC-SP-URC-${\mathrm{SBC}}_{\left[2,6\right]}^{*}$ scheme is unable to attain the said goal at any value of $E_{b}/N_{0}$, as shown in the trajectories of Figure 2.

**Figure 4:**
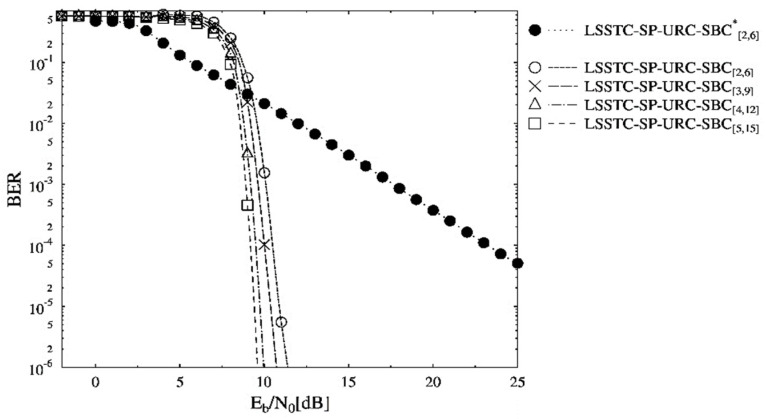
BER versus $E_{b}/N_{0}$ performance curves of the SBC schemes presented in Table 2.

The BER performance curves of the schemes presented in Table 2 are shown in Figure 4. The schemes in Table 2 are making use of the persistent overall code rate, but they differ only in the values of $d_{H,\min}$. It is clearly seen in Figure 4 that the BER curve associated with LSSTC-SP-URC-${\mathrm{SBC}}_{\left[5,15\right]}$ requires the least $E_{b}/N_{0}$ to reach the $10^{-4}$ BER value. Consequently, the BER performance of LSSTC-SP-URC-${\mathrm{SBC}}_{\left[5,15\right]}$ scheme is the best among others owing to the $d_{H,\min}$ value of 6, providing infinitesimal BER. The URC assists in attaining lower BER by distributing the information across the decoders and higher $d_{H,\min}$ values result in better distribution. Thus, the BER floor is not seen for higher than $10^{-4}$ values for the optimized SBC schemes. It is worthy to mention that the inner and outer iterations of the three-stage LSSTC system with ISCD become beneficial in acquiring the reliable LLR values. In other words, the information after the inner and outer iterations is refined enough to be more accurately fed to the SBC decoder for an enhanced BER. Moreover, the LSSTC-SP-URC-${\mathrm{SBC}}_{\left[2,6\right]}^{*}$ benchmarker offers the least BER performance as the $d_{H,\min}=1$ is unable to offer any iterative gain for EXIT convergence. More specifically, an $E_{b}/N_{0}$ gain of about 15 dB exists at the BER value of $10^{-4}$ for the LSSTC-SP-URC-${\mathrm{SBC}}_{\left[5,15\right]}$ scheme in comparison to the benchmarker.

**Figure 5:**
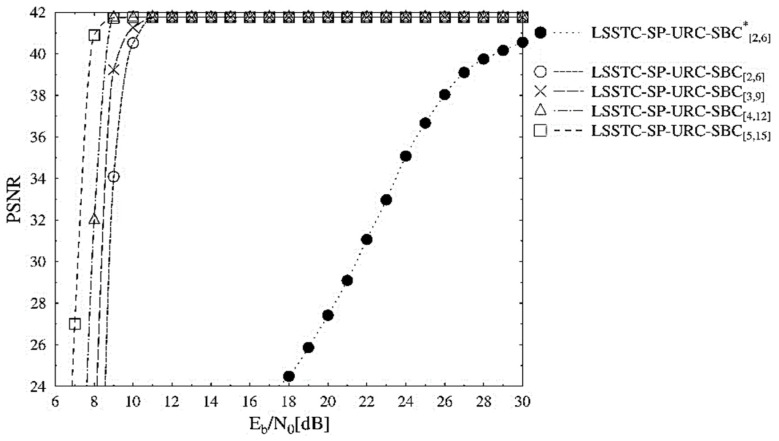
PSNR versus $E_{b}/N_{0}$ performance curves of the SBC schemes presented in Table 2.

Figure 5 depicts the PSNR performance trends of the schemes presented in Table 2. The PSNR performance curves also advocate for the LSSTC-SP-URC-${\mathrm{SBC}}_{\left[5,15\right]}$ scheme across the entire $E_{b}/N_{0}$ range. It is visible from Figure 5 that the LSSTC-SP-URC-${\mathrm{SBC}}_{\left[5,15\right]}$ outperforms the benchmarker by about 22 dB at the PSNR degradation point of 41 dB. The reason for the high gain in the PSNR is that we have employed a very compression efficient H.265 video codec, with the capability to reduce huge raw format video stream into considerably less amount of data. However, it is very important to note that the impact of transmission errors on the performance of decoded video stream becomes extremely gross on highly compressed video stream. This is due to the employment of advanced video coding techniques, such as variable length coding and predictive coding, resulting in the propagation of erroneous information into the neighboring pixels of the frame and even to the neighboring frames. Therefore, these errors result in a huge loss in the performance of the final decoded stream, with reference to the error free transmission.

**Figure 6:**
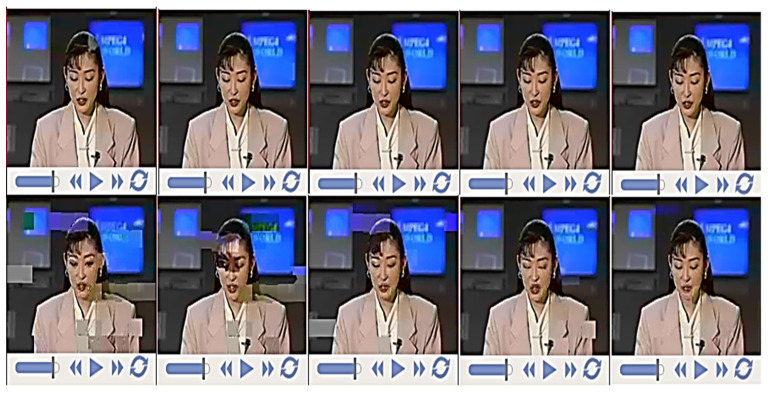
Subjective video quality performance using the (**top**) LSSTC-SP-URC-${\mathrm{SBC}}_{\left[2,6\right]}$ at (**left** to **right**) $E_{b}/N_{0}=$ 8.5 dB, 9 dB, 9.5 dB, 10 dB and 10.5 dB and (**bottom**) LSSTC-SP-URC-${\mathrm{SBC}}_{\left[2,6\right]}^{*}$ at (**left** to **right**) $E_{b}/N_{0}=$ 28.5 dB, 29 dB, 29.5 dB, 30 dB and 30.5 dB.

For the subjective video quality performance, the video sequences containing both the luminance and chrominance components were decoded via the H.265 decoder and the obtained quality is shown in Figure 6. This perceptual video quality indicator is clearly inclining towards the LSSTC-SP-URC-${\mathrm{SBC}}_{\left[2,6\right]}$ scheme over the LSSTC-SP-URC-${\mathrm{SBC}}_{\left[2,6\right]}^{*}$ benchmarker. More precisely, the optimized LSSTC-SP-URC-${\mathrm{SBC}}_{\left[2,6\right]}$ scheme attains much better video quality at lower $E_{b}/N_{0}$ values in comparison to the benchmarker. Distortions, even at the considerably higher $E_{b}/N_{0}$ value of 30 dB in the LSSTC-SP-URC-${\mathrm{SBC}}_{\left[2,6\right]}^{*}$ scheme, result in a substandard and defeated performance compared to the optimized LSSTC-SP-URC-${\mathrm{SBC}}_{\left[2,6\right]}$ scheme.

## 5. Future Work

In near future, we aim to incorporate the neural network and deep learning applications to multimedia streaming. More specifically, we aim to simulate the proposed system using convolutional neural networks (CNN) in order to further enhance the iterative decoding performance. The learning process for channel estimation and its behaviour can be devised by allowing the LLR values to the CNN along with the iterative decoding process. Moreover, we expect to extend the existing HEVC setup to low bit rate 3D and multi-view video. Furthermore, we plan to simulate our proposed three-stage system using the H.266 codec (developed in mid-2020) for supporting ultra-high-definition video streaming. Interestingly, the H.266 codec further compresses the data stream with the employment of sophisticated encoders and decoders.

## 6. Conclusions

This paper proposed an efficient three-stage system for avoiding the BER floor formation in most of the traditional two-stage systems. The design and performance curves for the three-stage wireless video communication system are provided. For efficient video transmission, we utilized the latest H.265 video codec. H.265 is widely adopted for achieving compression efficiency twice as much that of its precursor H.264 standard. The main constituents of the system were an SBC encoder, a URC precoder, and the modulation technique of SP. The URC assists in uniform distribution of information across the decoders. In addition, we investigated the performance of ISCD system with the combinational gain MIMO technique of LSSTC. More specifically, the system profited from the STC, V-BLAST and beamforming techniques. The system incorporated AAs for introducing transmit diversity gain. The BER and PSNR performances recorded significant improvements with the increase in $d_{H,\min}$ value. EXIT analysis verified BER improvement by the prevention of BER floor formation with the aid of optimized SBC for the three-stage system. Conclusively, an $E_{b}/N_{0}$ gain of about 22 dB is observed for the advocated system utilizing optimized SBC with $d_{H,\min}$ of 3 relative to the benchmarker with $d_{H,\min}$ of 1 at the PSNR degradation point of 41 dB. In future, we aim to incorporate deep learning-based CNN in the iterative decoding process to estimate the statistical behavior of the correlated Rayleigh fading model. Moreover, we aim to investigate the performance of the proposed three-stage system with the H.266 standard for ultra-high resolution (4 K to 16 K) video streaming.

## Figures and Tables

**Table 1:** An overview of major systems for wireless video communication.

References	Methods and Parameters	Video Coding Standard
[20]	Investigated the performance of SBC schemes using DSTS-SP for the two-stage system	H.264
[22]	Comparison of the regular and irregular error protection schemes using DSTS-SP for the two-stage system	H.264
[23]	Investigated the performance of Self-concatenated coding for the two-stage system	H.264
[24]	Comparison of convergent and non-convergent coding schemes for diverse video environments	H.265
[25]	Investigated the effects of varying $d_{H,min}$ on the BER and convergence performances for the two-stage system	H.264
[26]	Comparison of LDPC and polar codes for improvements in 5G networks	Not deployed
[27]	Presented a novel parameter for distortion measurement and offered a strategy to mitigate distortions	H.265
[28]	Systematic network coding and physical layer turbo coding for video transmission over satellite channel	H.265
Ours	Investigating low complexity SBCs with combinational gain technique of LSSTC for the three-stage system	H.265

**Table 2:** Rate-1/3 un-optimized and optimized SBC schemes with associated symbols and $d_{H,\min}$.

SBC Type	Symbols in Decimals	$d_{H,\min}$
Un-optimized ${\mathrm{SBC}}_{\left[2,6\right]}^{*}$	0, 16, 32, 48	1
${\mathrm{SBC}}_{\left[2,6\right]}$	0, 22, 41, 63	3
${\mathrm{SBC}}_{\left[3,9\right]}$	0, 78, 149, 219, 291, 365, 438, 504	4
${\mathrm{SBC}}_{\left[4,12\right]}$	0, 286, 557, 819, 1099, 1365, 1638, 1912, 2183, 2457, 2730, 2996, 3276, 3538, 3809, 4095	5
${\mathrm{SBC}}_{\left[5,15\right]}$	0, 1086, 2141, 3171, 4251, 5285, 6342, 7416, 8471, 9513, 10570, 11636, 12684, 13746, 14801, 15855, 16911, 17969, 19026, 20076, 21140, 22186, 23241, 24311, 25368, 26406, 27461, 28539, 29571, 30653, 31710, 32736	6

**Table 3:** Proposed system parameters.

Parameters	Value	Parameters	Value
Source Code	H.265/HEVC	Transmitter AAs	4
Source Bit-Rate	64 kbps	Receiver Antennas	4
Frame Rate	15 fps	Intermediate Code	URC
Slices per Frame	9	Interleaving Bits	10,000
Overall Code Rate	1/3	Video Sequence	QCIF Akiyo
Number of CTUs per slice	60	Normalized Doppler Frequency	0.01
Frames to be Encoded	300	Modulation Scheme	SP
Error Protection Scheme	SBC	Channel	Rayleigh Fading

## Data Availability

Not applicable.
